# Exploring the Nutrition-Related Health of the Ageing Population in Fiji: A Narrative Review

**DOI:** 10.1177/00469580241292433

**Published:** 2024-10-18

**Authors:** Salanieta Naliva, Marlena Kruger, Palatasa Havea, Carol Wham

**Affiliations:** 1 Massey University, Auckland, New Zealand; 2 Massey University, Palmerston North, New Zealand

**Keywords:** Fiji, nutrition, diet, health, older adults, aging

## Abstract

Fijis’ older population aged 55 years and over makes up 14% of the total population and is expected to reach 20% by 2050. This narrative review aimed to examine the health and nutrition status of the aging population of Fiji and sociodemographic determinants. A search strategy was conducted throughout databases, and gray literature from relevant websites was searched. Due to the limited evidence regarding the nutrition, health, and socio-economic factors that impact the aging population in Fiji the inclusion criteria were broad and included both genders (male and female), all publications up until December 2022, all study designs, and gray literature (government/institutional reports, conference proceedings, guidelines, Act, and Policies) . There was no filter for date applied in the search criteria. Studies that did not meet the search criteria were excluded. 20 documents including published articles were included for analysis and result synthesis. Life expectancy at birth for the Fiji population is 68 years. A significant annual increase in mortality rate from endocrine, nutritional, and metabolic diseases has been observed in women aged 75+ but not older men. Women of low-income status are more at risk than men. However, as most investigations aggregate those ≥18 years, there is a lack of information on older adults (≥65 years) health and nutrition status. To improve the health status of older adults, an understanding of the nutritional status of older adults is warranted, especially concerning lifestyle and sociodemographic determinants.

**What do we already know about this topic?**
The aging population is expected to reach nearly 20% of the total population in 2050. Parallel to the rapid growth of the older population in Fiji is the increasing prevalence of non-communicable diseases. Government efforts to address the aging population have mainly focused on social and welfare protection with little emphasis on health and wellbeing.
**How does your research contribute to the field?**
A sociodemographic overview of the older adult population in Fiji and what legislation and policies are currently available for social welfare protection and wellbeing. The importance of underlying physiological, socio-economic, and psychological factors influencing health and nutrition status.
**What are your research’s implications towards theory, practice, or policy?**
This review aims to explore the nutrition-related health of the aging population in Fiji. Understanding the context of the aging population in Fiji, and the socio-economic factors that impede health status can help establish best evidence practices for nutritional interventions to improve health and well-being of the older populations in Fiji.

## Introduction

Population growth is expanding globally, with more people expected to live longer and beyond their sixties. This is evident across the globe as most countries are experiencing growth in both the size and proportion of older persons in the population. The global forecast anticipates that by 2030, 1 in 6 people will be 60 years or over. The number of persons aged 80 or older is expected to triple between 2020 and 2050 to reach 426 million.^1^ The population of the Asia-Pacific region is aging more rapidly than any other region, especially in Fiji, where the population over 60 years is expected to triple between 2000 and 2050.^2^

**Figure 1.**
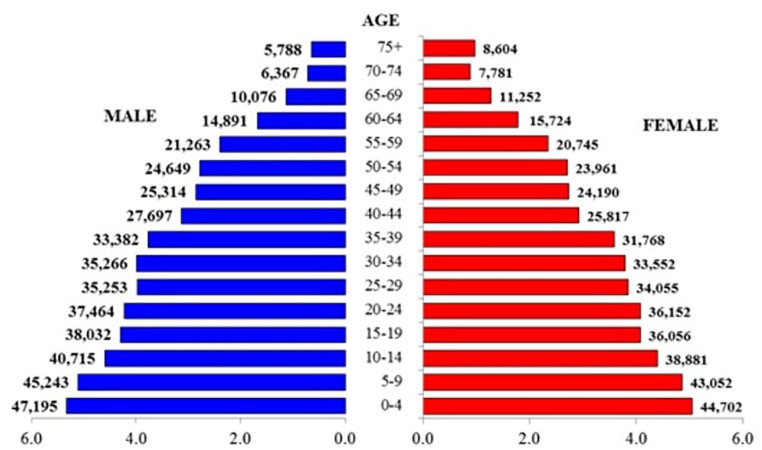
Population Pyramid showing Fiji’s age-sex structure.^2^

Fiji is the second most populated of the Pacific Island countries (PICs), with a population of 884 887 at the 2017 census, of which 55.9% reside in urban areas while 44.1% are in rural areas.^2^ Besides the increasing population, Fiji is among the most rapidly aging Pacific Island countries. In 2017, 14% of the population (122 000 persons) were aged 55 years and over.^3^ Figure 1 shows Fiji’s age-sex structure as of Census Night 2017. The broad base of the system indicates a young population. The median age of Fiji’s population is 27.5 years which means that half of Fiji’s population is below that age. The predominant ethnic groups in Fiji are 60% i-Taukei (indigenous) Fijians; and 34% Fijians of Indian descent, with 6% comprised of Pacific Islanders, Asians, Europeans, and others.^2^

**Figure 2.**
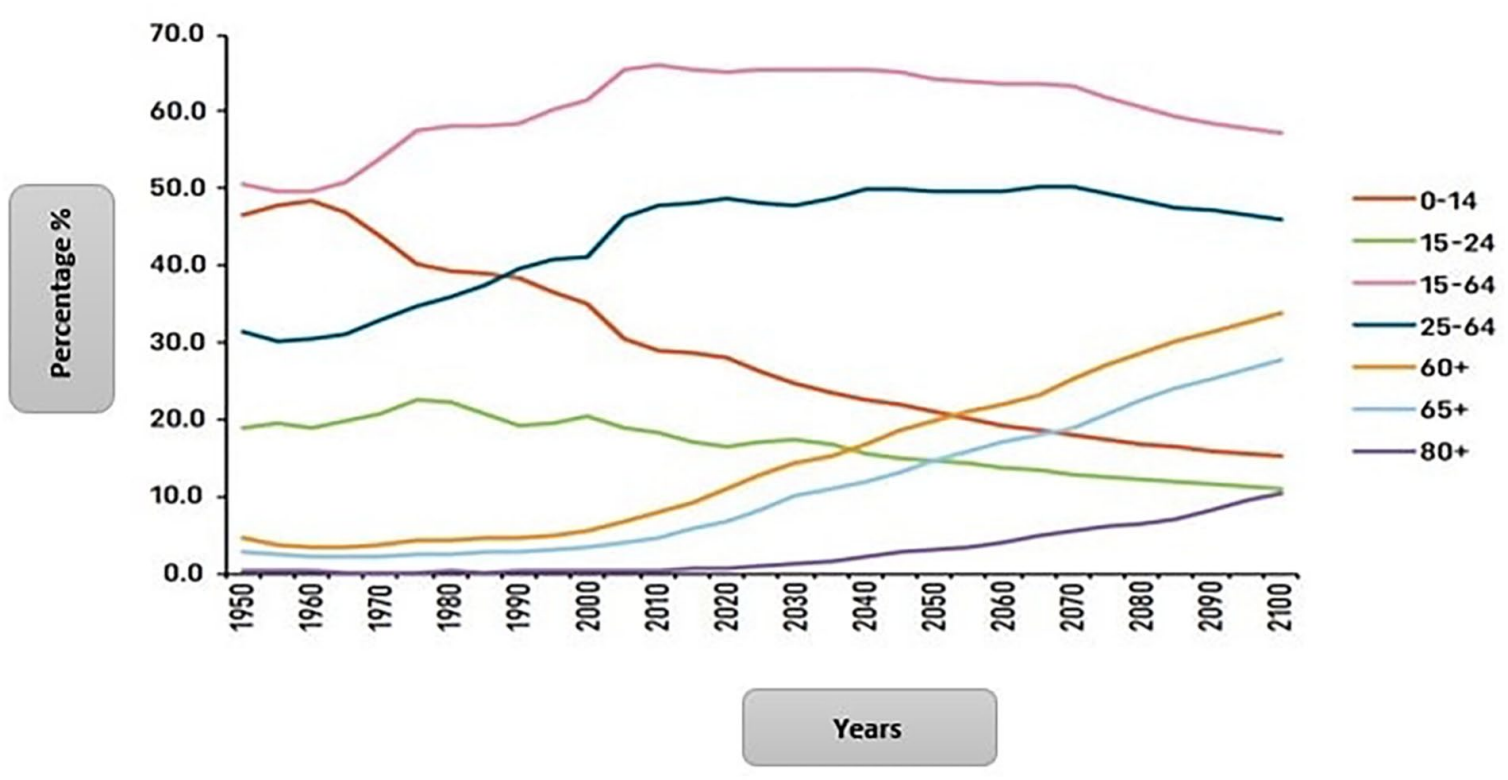
Trends in percentage population cohorts in Fiji, 1950-2010.^4^

Although the population of Fiji is relatively “young,” demographic changes such as rapid declines in fertility and increased life expectancy are leading to profound population shifts. In comparison, the percentage of the older population aged 60+, as shown in Figure 2, has been increasing steadily since 2000 and is expected to reach nearly 20% of the total population in 2050.^4^ By 2050, the female population aged 60+ is projected to reach 22.3% of the entire female population, whereas it will be 17.5% for males. Globally, the higher number or proportion of older women to older men in the population is a common phenomenon, given that women have a longer life expectancy than men.^5^

Although there are commonly used definitions of old age, there is no general agreement on the age at which a person becomes old. Families and communities often use other socio-cultural referents to define age, including family status (grandparents), physical appearance, or age-related health conditions. The United Nations defines an older person as those aged 60 years or over. On many occasions, it is defined as 65+.^6^ The Fiji National Policy on Aging 2011-2015 described being old as any person aged 60 years and over.^7^ Parallel to the rapid growth of the older population in Fiji, the non-communicable diseases (NCDs) “crisis” has created further challenges for health systems. NCDs contribute to 80% of all deaths in Fiji with hypertension and diabetes among the top 5 causes of death. “Premature death,” that is, dying early before 60 years old is common in Fiji, with more than fifty percent of males dying earlier than females between the ages 45 to 59 years.^8^

Older adults are faced with challenges that impact food intake and their nutritional status, such as physiological changes that affect functional abilities,^9-11^ psychosocial burdens such as social isolation, loneliness, and depression,^12-14^ economic instability such as the unavailability of finances^15-17^ and other interwoven determinants such as frailty, hospitalization, and polypharmacy that may generally contribute to a person’s nutrition risk status.^18^ In Fiji, social protection is provided firstly by the safety net of the extended family with the pension scheme at age 55 being inadequate for those living alone.^19^ In this case, most older adults cohabitate with their children, children’s families, or other relatives. Communal living with extended families is common. Having low income to sustain everyone in extended living can be a burden and resources such as food, medicinal purposes, and travel to hospitals may be spent on other family members rather than the older adult member. For this reason, it is important to understand the socio-economic context that an older Fijian adult may live in.

Despite the established and growing evidence of diet-related diseases and conditions and the importance of diet across populations in Fiji,^20-23^ there is inadequate evidence about what older adults are eating and what factors influence their ability to live better and longer. A few studies have attempted to shed light on the health and socio-economic issues impacting older adults in Fiji,^19,24-29^ interestingly Seniloli and Tawake,^19^ found that 27% of older adults in Fiji have impaired health status (disability), however, none have captured the dietary intake and nutrition status of this population. This narrative review aimed to explore existing Act and Policies and describe the nutrition-related health of the aging population in Fiji.

## Methods

This review seeks to answer the following question:

### What Are the Socioeconomic Contexts That May Affect the Nutrition-Related Health of Older Fijians?

The articles were searched on the Massey University library website. Databases like Web of Science, Research Gate, PubMed, Scopus, and Google Scholar were used. The free web-search route, Google Scholar and the gray literature from World Health Organization, UNICEF, Fiji Women’s Crisis Center, Fiji Ministry of Health and Medical Services and the Fiji Government using the web url.gov.fj were also searched. The search terms included derivatives of critical key phrases such as nutriti* OR “nutritional status*” OR diet* OR “food intake*” AND aging OR “older adult*” OR “55+ year*” OR “older people*” OR “older person*” OR “senior adult*” OR geriatric* OR elder* AND Fiji* AND health*. The titles and abstracts were screened. Full texts of eligible articles in English were retrieved.

The inclusion criteria were both genders (male and female), all publications up until December 2022, all research with different study designs, and gray literature (government/institutional reports, conference proceedings, guidelines, Act, and Policies) were considered in this review due to the limited available evidence from Fiji regarding the nutrition, health and socio-economic factors that impact the aging population. There was no filter for date applied in the search criteria. Studies that did not meet the search criteria were excluded. 20 documents including published articles (13),^19-23,25-32^ government reports (5),^33-37^ an unpublished thesis (1),^38^ and a book (1)^24^ were included for analysis and result synthesis.

## Review

### Current Acts, Policies and Programs, and Reports Addressing Population Ageing in Fiji

*i. National Council for Older Persons (NCOP) Act 2012*

The Fiji Government has passed legislation that promotes and addresses the well-being of older persons. *The National Council for Older Persons Act 2012*, was promulgated as Decree No 63 of 2012 and commenced on 30 October 2012.^39^ This legislation led to the establishment of the National Councils for Older Persons aimed at promoting inclusivity, instilling dignity, and respect for human rights, and meeting basic needs through the empowerment of older persons and related matters. As a Council, NCOP was directly aligned to the National Aging Policy [2011-2015]. The council also served as the umbrella body of all services and programs of older persons, and any organizations that provide such services will need to be registered under the Council.^38^

*ii. National Council of Older Persons*

As an advisory body for the Government on matters relating to the rights and protection of older persons, the Ministry of Women, Children, and Poverty Alleviation has progressively created awareness and undertaken consultations with older persons themselves to identify their needs and how these can be addressed. In line with its focus to ensure better coordinated efforts to address issues affecting older persons, the government continues with its work to formalize district committees for older Persons. The council was established in 2013^40^ to promote the care and welfare of the older population in Fiji. This advisory body is therefore responsible for issues such as finance and social and welfare protection.

*iii. Fiji National Policy on Aging 2011 to 2015*

The National policy on aging was developed in 2011 through a collaborative effort between the Ministry of Social Welfare, Women and Poverty Alleviation and the United Nations Population Fund (UNFPA) Pacific Sub-Regional Office. The objectives of the policy are the inclusion of older people in the community, their health and welfare, and the extent to which they can support themselves within the community specifically on Goal 3: Healthy living. This aims to improve the overall health of older persons, firstly to integrate provisions for older persons in all health sector planning and secondly, to strengthen primary healthcare services to meet the needs of older persons.

*iv. Minimum Standards for Residential Homes for the Elderly*

In the institutional Aged Care settings, the Government 2017 established a National Minimum standard for Aged Care Facilities in Fiji. The standards aim to address the minimum requirements for facilities to operate as care homes. This included the required knowledge, skills, and competencies needed by the management and staff to ensure that care homes deliver individually tailored, comprehensive, and quality services.^40^

*v. Women and Aging: Scoping Study on Perceptions of Aging Among Women in Fiji*

This was the first-ever scoping study completed in Fiji to explore views about women and aging more generally. The findings provide a snapshot of attitudes and perspectives surrounding the “feminization” of aging, which shows that more women are aging than men. Globally, it is projected that women will live longer than men, and this trend is expected to remain unchanged until 2050.^41^ Like women of all ages, older women actively contribute to society and lead diverse, and vibrant lives. However, women tend to disproportionately face challenges in terms of financial security and healthcare access. Participants of the study agreed that the formal retirement age should revert to 60 years from the current 55 years. Poverty was also identified as directly linked to aging, with older women not having an income base, nor entrepreneurially inclined.

*vi. Aging Women and Poverty in Fiji: A Preliminary Review of Global to National Commitments*

The challenges and barriers experienced by women as they age are interwoven. Older women in developing countries, such as the Pacific Island region, experience higher economic dependency on men because of existing customary laws that restrict access and ownership of land, resources, financial autonomy, and other assets.^35^ The report also mentioned that in some countries in the Pacific Island region, older women are left widowed and do not have the support of a spouse, thus increasing their vulnerabilities. For example, in Fiji, many women earn less and are employed in more insecure jobs than men. As a result, many older women need more financial resources when they retire.

*vii. Health and Aging Report*

Improving the health and wellbeing of older Fijians is a national health priority. In Fiji, the ability of health system planners to identify and utilize appropriate and effective models of care to support healthy aging has been hindered by a lack of knowledge of what is needed and what works better for the care of older persons in the local context. The joint research effort by the health ministry and several institutions generated valuable findings to guide an effective health system response to population aging.^36^

*viii. Income Security for Older Persons in Fiji*

Universal access to a pension is important for the health and wellbeing of the aging population. This, however, requires the workforce to have employment and save throughout their working life. The Income Security report documented existing income security schemes for older persons in Asia and the Pacific, including Fiji, and identified areas needing reform.^42^ Fiji’s aging population demography poses challenges to achieving income security and adequate social protection for older persons. The report examined the current government programs on the social security systems such as the family assistance program (FAP), poverty benefit scheme (PBS) and the superannuation funds scheme which is the Fiji National Provident Fund (FNPF). Findings showed that despite the range of pension options offered by FNPF, most members choose a lump sum payment rather than a life or fixed term annuity with over 80% of members who were retiring in the following years only had a balance of FJD 50 000 or less. Alarmingly, this amount is well below the threshold required to sustain them during old age from when they retire at 55 years.

### Health and Aging

Population aging presents significant implications for economic, health systems, and social protection globally. More so, understanding that the aging process does not always follow a linear pathway can present challenging health issues. The Healthy Aging -Fiji study is the only study that discussed the health status of older adults.^36^ Circulatory system diseases were the most common reason for hospital admissions and outpatient visits and older men and women aged 75+ experienced a significant annual increase in mortality rates from endocrine, nutritional, and metabolic diseases as compared to those in the lower category of older adults.^36^ Moreover, understanding the needs of older adults, caregivers, and service providers is paramount. Research by Palagyi et al noted that older adults tend to be concentrated in a few areas within each country, some in the maritime zones (islands), rural areas while majority remains in the urban centers. This means that a few regions will face specific social and economic challenges due to population aging. These demographic trends have several implications for government and private spending on pensions, health care, education, and economic growth and welfare.^6^ For example, rural dwellers are isolated and tend to migrate to urban areas simply because no health services are available. Hence, there is a strong preference for comprehensive community-based care models that enhance access of older adults to health services.^36^

### Health and Functional Abilities

*a. Physiological factors*

As older adults age, physical strength, functional ability, and capacity to do normal activities of daily living decline. Problems such as poor eyesight, difficulty chewing, and foot problems were some physiological factors that affected the health of older persons.^19,24^ Older adults would participate daily to function ordinarily, such as handling money, getting dressed, getting in and out of bed, walking, taking a bath, and visiting the toilet in time as well as taking adequate care of one’s appearance. A high number (80%) of the participants reported that they could do the necessary activities.^24^

The measurements of disability in older adults continue to attract interest in studying an aging population and how it impacts their quality of life and longevity. Disability in any form can impact a person’s daily activity routine. Having some form of disability in older adults diminishes their ability to function and poses challenges to their health. A disability risk assessment for older adults showed that 10% of the aging population experienced a disabling condition, which is most prevalent among older widowed women and married older men in rural areas.^27^ Similar findings in another study noted that older females were more susceptible to a disability than males.^19^

NCDs have exacerbated the capacity of older adults to function daily such as walking, chewing and being able to see. While age was a significant predictor of disability amongst the older people in Fiji, several older adults also reported that their disability had been fueled by chronic diseases such as diabetes and arthritis, with approximately 47% of those surveyed indicating that they had at least one chronic illness.^19^

*b. Socio-economic factors*

In the Pacific, the extended family is the main provider of care and social security for the aging population. Living arrangements also impact the wellbeing of older adults. As age increases, most older persons live with a family member, especially with their children and grandchildren with only few living alone.^24^ Similar findings were noted in a study that as age increases, the proportion of older adults living alone declines, and co-residency increases.^19^ Marital status of older adults was also important in assessing their living arrangements. Fewer older adults lived with their spouses, and widowhood was more predominant among women than men, with 12% only of women living with spouses ≥80 years compared to 64% of males.^24^ Sex and age were also indicative of the living arrangements of older adults, where males and the young-old were generally more likely to live with their children than females or the old-old.^30^

Ethnicity also has an influence on the living arrangements of older adults in Fiji. Important to note that indigenous Fijian families are less likely to send their older family member or ill family members to a facility to be cared for. In addition, having another person who is not a family member or immediate relative to care for older family member is seen as a social disgrace and an irresponsibility act of the children. It is common to see immediate family members care for their loved ones until they can no longer do so in the family home. On the other hand, Indo-Fijians were most likely to live alone compared to ethnic Fijians.^19^

Co-residence is common, especially in urban areas where the cost of living is high. In general, co-residence is still common among most older adults, given the decline in their financial status on one hand and children being dependent on their older parents on the other. Retirement plans without savings and future financial security is the leading cause of cohabitation. Most of the older adults in Fiji are not able to provide accommodation and economic support for themselves in old age and that older adults living with their children, family members, and relatives are most likely to have health ailments.^19^

### Psychological Factors

As age increases, the ability to think and utilize brain functions, deteriorates. Mental health issues in older adults have been established and cognitive decline are common. Forgetfulness, loss of memory and interest and fatigue were identified in older adults but more in males than females.^24^ Similarly, loneliness and isolation were identified as detrimental to their well-being. Psychological decline is exaggerated by the loss of a spouse, migration of children, and absence of peers which triggers feelings of loneliness and isolation.^24^ Overall, loneliness was correlated highly with other factors like economic circumstances, socio-economic status, and marital status. For example, lack of money to see friends and family is associated with feelings of loneliness.

### Dietary Intake and Nutrition Status

The dietary intake and overall nutrition status of the adult population ≥ 18 years have been documented in a small sample of the adult population,^31^ national nutrition survey,^33,34^ NCDs risk factors survey,^43^ and dietary diversity study^37^; however, there is an absence of knowledge on the dietary intake and nutrition health-related status of older adults. Findings of a small sample population highlighted that 83.4% of those screened were overweight and obese and it was common among female I-Taukei indigenous Fijians aged 40-61.^31^ High blood pressure was more prevalent common among the population compared to high blood sugar.

Data from the Fiji 2011 STEPS survey,^43^ showed that consumption of fruit and vegetables (mean servings a day) was generally low, with a mean of 1.2 servings of fruit and 1.9 servings of vegetables. This meant that 85% of the population did not meet the recommended 5+ servings of fruit and vegetables a day. Indo-Fijians generally consumed more fruits and vegetables than iTaukei indigenous Fijians, with minimal differences by age or gender. In addition, there has been a significant increase in levels of overweight and obesity in Fiji, with significantly more iTaukei women found to be obese (42.0%) than men (22.4%).

Dietary diversity data were collected from 369 participants and classified into food groups. Proportions of intake per food group at household and individual levels showed that refined grains (cereals) were the most consumed food group (80%).^37^ Most participants reported growing some of their food (n = 384; 82.2%). The 5 most frequently reported items were cassava (62%), eggplant (54%), bele (49%), beans (34%), and taro (34%). Similarly, an assessment of food intake in a rural village in Fiji showed that the 2 main foods eaten were cassava and flour based goods.^32^

The Fiji National Nutrition Surveys of 2004 and 2015^33,34^ for adults ≥ 18 years using a 24-h diet recall showed daily energy contributions were 15% from protein, 27.8% from fat, and 53.4% from carbohydrates. In contrast, in 2015, protein, fat, and carbohydrate contributed 17%, 27%, and 56% of daily energy, respectively. Consistently, more than 50% of those surveyed did not meet the recommended intake for dietary fiber, Vitamin A, iron, and calcium. Primary food sources of protein were fish, meat, and poultry. Fats include vegetable oils, margarine, whereas carbohydrate sources were from traditional staples, rice, and flour products. Being overweight and obese are of significant concern as their prevalence increased from 32.3% in 2004 to 63.5% in 2015. Similarly, anemia prevalence increased from 32.4% to 40.1% from 2004 to 2015.

Despite the current knowledge of the adult population’s dietary intake and health status in Fiji, both the Fiji National Nutrition Surveys of 2004 and 2015 aggregated all adults ≥55 years; therefore, the nutrition status of older adults is unknown.

## Discussion

This narrative review explored the nutrition-related health of the aging population in Fiji. Using a range of academic databases, the review found few population aging studies to provide a solid basis for evidence-based policy and intervention. The demographics of this population are forecasted to increase to 20% by 2050,^3,4^ therefore planning for the aging population is critical. The National Council for Older Persons Act 2012 is the current legislative document that overarches every other policy and program mandated in the country for the care of older persons. Most of these legal documents have existed for over a decade, and revisions are greatly needed to align with current global population aging efforts.

Few scholars^19,24,27,29^ have dedicated their research from the late 1980s to mid-2000s to health and socio-economic factors affecting this vulnerable population. The findings of this review included firstly the living arrangements of older adults where most older adults cohabitate with their children, children’s families, or other relatives. Communal living with extended families is common for Indigenous Fijians and not Fijians of Indian ethnicity. Secondly, low socio-economic status also affects their livelihood. The burden of low income in extended living to sustain everyone in the household may pose nutrition and other health risks for older adults.

Thirdly, psychological factors such as loneliness, memory loss, and isolation affect their nutrition status and vice versa. Fourthly, an overview of the nutrition status of the adult population found that most studies combined all adults ≥18 years. For instance, the Fiji National Nutrition Surveys of 2004 and 2015 both reported that more than 50% did not meet the recommended intake of dietary fiber, Vitamin A, Iron, and Calcium.^33,34^ however, the older adult’s nutrition status is largely unknown. Lastly, a recent study assessed existing national health policies, programs, and services supporting healthy aging which was also the first for any Pacific Island nation.^36^ The report identified existing health system barriers to healthy aging in Fiji and provided recommendations to improve the health and well-being of older adults. It is hoped that these recommendations would support not only the Ministry of Health and Medical Services but contribute to a broader multi-sectoral approach to population aging.

Findings from this study showed that Circulatory system diseases were the most common reason for hospital admissions and outpatient visits and older men and women aged 75+ experienced a significant annual increase in mortality rates from endocrine, nutritional, and metabolic diseases. In addition, visual and hearing disabilities, diabetes, and hypertension are common among both urban and rural older adults in Fiji.^36^ Importantly, geographical locations may also have implications for the distribution of health services to older adults. Older adults seem to return to their home community during their later years to reside, placing added responsibility on family and carers who are often not well-resourced to support their health care needs. Despite the government’s efforts to decentralize health services, older adults living in rural and maritime (islands) are at a disadvantage in assessing the health services they need, when they need them. The frequency of outreach programs with limited resources and the lack of gerontological knowledge and skills of health workers may contribute to the disparity of health services in geographical locations.

## Strengths and Limitations

To the author’s knowledge, this is the first broad overview of Fiji’s nutrition-related health of older adults. This review included a comprehensive search of the literature using multiple databases to ensure relevant research papers were captured. The narrative review introduced the demography of the aging population of Fiji and explored existing legislation, policies, programs, and reports that address issues faced by the aging population, including the health and aging background of this population. Moreover, nutrition-related health factors such as physical, socio-economic, and psychological ones play essential roles in the well-being of older adults.

There are several limitations to this review. A systematic approach to the literature search was not undertaken to include the limited available evidence within Fiji. In addition, only dietary intake of the adult population ≥18 years old was observed for all dietary intake studies and reports. There is undoubtedly a gap in knowledge of understanding older adults’ dietary intake. Despite this, the review provided a breadth of current evidence on the nutrition-related health factors of the aging population in Fiji. The literature search was guided by the paper’s authors, who have done extensive work on the social aspects of aging in Fiji, co-authors of this current paper, and the expertise of a librarian was utilized to reduce the risk of bias. The search was restricted to English-language publications. Further literature searches outside of Fiji were not conducted to keep within the scope of this paper.

## Conclusion

It is important to understand the socio-economic context of an older adult. In Fiji, these socio-economic factors may have implications for the nutrition status of older adults. Older Indigenous Fijian women, with low income are more vulnerable than men. This review has explored the nutrition-related health of the aging population that will add to research knowledge of the aging population and could also contribute to the policy and decision-making of developing countries like Fiji.
